# Cultivation viability of *Allium tuberosum* L. in the Western Ghats: insights into crop dynamics, yield and quality

**DOI:** 10.3389/fpls.2024.1480510

**Published:** 2024-11-18

**Authors:** Abhishek D. Gavhane, Rajiv B. Kale, Yogesh Khade, Hem Raj Bhandari, Shivam Y. Gaikwad, Sharadveer Singh, Ahammed Shabeer T.P., Yogesh A. Garde, Kiran Khandagale, Vijay Mahajan

**Affiliations:** ^1^ ICAR-Directorate of Onion and Garlic Research, Pune, India; ^2^ ICAR-National Research Centre for Grapes, Pune, India; ^3^ N.M. College of Agriculture, Navsari Agricultural University, Navsari, India

**Keywords:** underutilized *Allium*, non-traditional cultivation, yield optimization, seasonal effects, Western Ghats agro-climatic region

## Abstract

*Allium tuberosum*, commonly known as garlic chives, is an underutilized *Allium* species despite its significant culinary value for its mild garlic flavor and therapeutic potential due to the presence of sulphur-containing compounds with antimicrobial, anti-inflammatory, and antioxidant properties. This study assessed the cultivation potential of *A. tuberosum* in the non-traditional agro-climatic region of the Western Ghats, focusing on the effects of cultivars and seasonal variations on growth, yield, and quality in two-year field trials. Among the accessions tested, *A.tuberosum* All-1587 demonstrated the highest yield, producing 157.01 tons of green foliage per hectare. Bright sunshine seasons positively influenced both yield and quality, while the monsoon season induced morphological changes such as increased stem length, reduced leaf width, and decreased stem girth, traits generally considered inferior for market quality. The monsoon also led to increased waste generation, highlighting the need for careful management during this period. Nutritional analysis revealed high concentrations of potassium (5355 mg/kg), phosphorus (691 mg/kg), and sulphur (2484 mg/kg), while biochemical profiling identified bioactive compounds such as flavonoids (3.19 mg/g) and organosulfur compounds, including Allyl Methyl Thiosulfinate (269.00 mg/kg), which contribute to the plant’s notable health benefits. These findings support the suitability of *A. tuberosum* for year-round cultivation in the Western Ghats and its potential for commercialization, especially in regions with similar climatic conditions.

## 1 Introduction

The genus *Allium* characterized by its distinctive scented underground bulb is one of the largest plant genera comprising nearly 1000 species with *Allium cepa* (onion) and *Allium sativum* (garlic) being commercially important crops (Ochar and Kim, 2023). The genus includes some underutilized and semi-domesticated species which are equally potential for commercialization. These species have significant culinary uses, primarily adding flavor and savor to food. The flavor arises from the chemical transformation of S-alk(en)yl cysteine sulfoxides into volatile compounds, including S-methyl cysteine sulfoxide, S-allyl cysteine sulfoxide, and S-trans-prop-1-enyl cysteine sulfoxide, among others (Jones et al., 2004). Despite multifaceted uses, many species within the genus remain largely underutilized, which is a missed opportunity considering their importance for food and medicinal properties as alliums are rich in secondary metabolites like organosulfur compounds, flavonoids, phenols, saponins, alkaloids, and polysaccharides. By virtue of this, they offer a range of nutritional, biological, and health benefits, such as antimicrobial, antioxidant, antitumor, immunomodulatory, antidiabetic, and anti-inflammatory properties (Riaz et al., 2020; Yan et al., 2022; Kim et al., 2023). Processed products in the form of dried bulbs, leaves, buds and flowers from wild *Allium* species like *A. tuberosum, A. angulsorum, A. auriculatum, A. carolinianum, A. griffithianum, A. hummile, A. roylei, and A. wallichii* are in demand for their culinary applications and extended shelf life (Negi et al., 2006). 

*A.tuberosum* commonly known as garlic chives, Chinese chives, or Oriental garlic, remains an underutilized species despite its substantial culinary and medicinal applications. Native to Southeast Asia and often cultivated in home gardens in northeast India (Pandey et al., 2014), this perennial species thrives in moderate climates and is particularly valued for its mild garlic flavor due to the presence of methiin, alliin other sulfur compounds (Xia et al., 2022). It is especially useful in dishes where raw garlic would be too overpowering, such as salads, egg dishes, soups, stews, stir-fries etc. The flowers are also edible and can be used as a garnish. In several countries including China, Philippines, Korea, and Thailand, garlic chives are a well-known vegetable (Saito, 2020; Alam et al., 2023.

Past research on *A. tuberosum* has revealed a rich profile of essential micronutrients and vitamins vital for various bodily functions, including immune response, bone health, and red blood cell formation (Bastaki et al., 2021). Medicinally, *A. tuberosum* seed extract is valued for its aphrodisiac properties (Guohua et al., 2009). The presence of organosulfur compounds, similar to those found in garlic, enhances its therapeutic potential by providing antimicrobial (Khalid et al., 2014), anti-inflammatory, anti-cancer (Park et al., 2013), antidiabetic benefits (Tang et al., 2017) etc.

Field surveys and research have highlighted the edible uses of *A. tuberosum* in the Himalayas, particularly in the Garhwal and Kumaon regions (Joseph and Pradheep, 2019). *A. tuberosum* consumed as raw or cooked vegetables, spices, and seasonings. Beyond these primary regions, it is gaining importance in the plains and other non-traditional areas, serving as an alternative to leaf-purpose onion cultivation, which faces challenges like susceptibility to biotic and abiotic stresses and seasonal availability cycles (Huang et al., 2016; Khandagale et al., 2022). Pandey et al. (2014) stated that *A. tuberosum* has the potential for commercial cultivation in different onion and garlic growing part of the India. The ICAR-DOGR in Pune has been working on primary studies for *A. tuberosum* in this new geographical setup to cater for a year-round supply and developed a value chain model (Kale et al., 2024). Recent agronomic research has focused on improving the cultivation practices of *A. tuberosum* to enhance its productivity and quality. Studies have found that the use of slow-release fertilizers and mulching increased the growth, nutrient use efficiency, weed control, yield and biological value of *A. tuberosum* (Wang et al., 2020; Adamczewska-Sowinska and Turczuk, 2016; Kim, 2010).

The economic importance of *A. tuberosum* extends beyond its culinary and medicinal uses. The plant has significant market value due to its increasing popularity in gourmet cuisine and traditional medicine (Kalasee and Mittraparp-arthorn, 2023; Alam et al., 2023). The demand for fresh and processed products example dried leaves and flower buds, is rising globally, driven by consumer preferences for natural and health-promoting foods (Imahori, 2022; Liu et al., 2021; Kale et al., 2024b). Its unique flavor due to the presence of sulfur-containing volatiles such as Diallyl disulfides (Xia et al., 2022) and health benefits like antidiabetic, hepatoprotective, and antitumor nature (Tang et al., 2017) make it a sought-after ingredient in high-end restaurants and health food stores. Additionally, the development of value-added products including powder and feed capsules, is opening new avenues for commercial explorations (Annual report ICAR-DOGR, 2021). Western Ghats, characterized by its climatic variability and ecological diversity, offer a promising environment for agricultural diversification, particularly in response to the limitations faced by traditional crops such as onions and garlic, which are prone to biotic and abiotic stresses (Huang et al., 2016). This study aims to assess the adaptability of *Allium tuberosum* in this ecologically unique region, focusing on developing sustainable cultivation practices that can enhance agricultural diversity and resilience. Given that the Western Ghats present a warmer climate compared to the native habitat of *A. tuberosum*, this research seeks to explore the potential of year-round cultivation, overcoming the seasonal constraints imposed by harsh winters.

In light of significant agronomic variations, such as sowing methods (either from seed or bulb), planting layouts, and intercultural operations, this study will establish foundational guidelines for optimizing these practices in the Western Ghats. The primary objective is to evaluate the cultivation viability and nutritional profile of *A. tuberosum* in non-traditional geographical regions, with an emphasis on the Western Ghats. By analyzing its adaptability to novel environmental conditions, this research aims to provide a comprehensive framework for popularizing *A. tuberosum*, contributing to agricultural diversification and nutritional security. Furthermore, the study seeks to identify commercial opportunities for *A. tuberosum* within the expanding health food market, offering farmers and agribusinesses access to a profitable and emerging agricultural niche.

## 2 Materials and methods

The *Allium* germplasm in the collection was cultivated in normal conditions of western ghats to assess their production performance in the region. Three accessions viz. *A.tuberosum* All-1587, *A. tuberosum* CGN-116418 & *A. tuberosum* CGN-16373 referred to as AT1, AT2 & AT3 respectively for convenience, were used in the present study for production performance and assessment of nutraceutical properties. The nutritional and biochemical profile of the best-suited *A. tuberosum* line was done to assist the commercialization by advocating its health and related benefits.

### 2.1 Location, soil and general weather conditions

**Figure 1:**
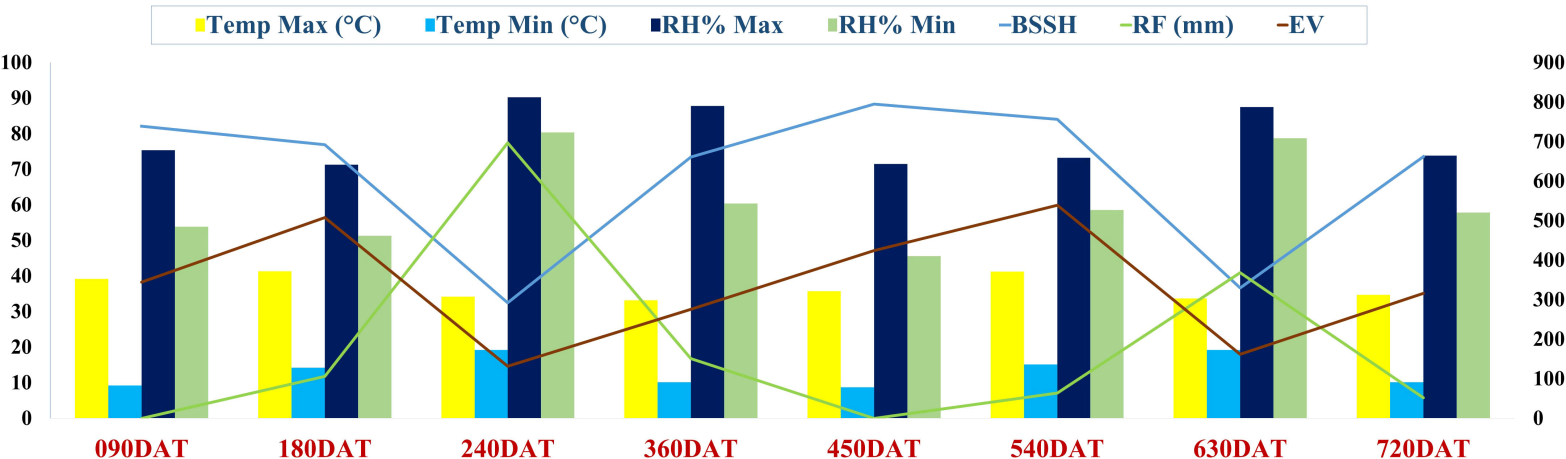
General weather conditions during the experiment period.

The study was conducted at the ICAR-Directorate of Onion and Garlic Research (ICAR-DOGR), Rajgurunagar, India. The region falls under the Western Maharashtra Plain Zone agroclimatic zone as per the classification by ICAR (Ahmad et al., 2017). This is a region of the regur (black) soil. Wheat, gram, millets, cotton, pulses, groundnut, and oilseeds are the main crops in the rain-fed areas, while in the irrigated areas, sugarcane, onion and wheat, are cultivated. The experimental site is situated at 18° 50’ 33.5112’’ N and 73°53’5.7732’’ E with an elevation of 553.8 m above MSL. The average annual temperature ranges from 5.5°C to 42.0°C, while the average annual rainfall is 669 mm. The predominant soil type at the site is black vertisol with a pH of 7.27, an electrical conductivity of 0.26 dS/m, and a soil organic carbon (SOC) content of 6.98 g/kg. The available nutrient status of the soil is favorable for crop growth, with nitrogen at 219.82 kg/ha, phosphorus at 52.82 kg/ha, potassium at 824.0 kg/ha and sulphur at 28.54 kg/ha. The general weather conditions prevailing during the experiment period are depicted in **Figure 1**.

### 2.2 Selection of tubers, planting and general operations

Three *A. tuberosum* accessions were evaluated employing a factorial random block design. The accessions of *A. tuberosum*, constituted one factor, while eight age windows each comprising a three-month unit, formed the second factor. The selected planting material i.e. rhizomes from the three-year-old mother batch were carefully extracted and separated from the clump. No treatment was applied to the rhizomes, which were then planted at 25×25cm spacing on a raised bed of 1.2 m width and 15 cm height. Five replicates of each *A. tuberosum* line were planted, with each replicate consisting of 35 hills arranged in seven rows of five hills each. Two drip laterals with emitters spaced 30 cm apart were installed over the bed for irrigation. Weeding was the primary interculture operation in the research field. According to the age windows, the third, fourth, sixth, and eighth windows exhibited higher weed infestation rates, necessitating frequent weeding. Irrigation scheduling was managed based on field moisture conditions, with the second, third, fifth, and sixth age windows requiring less frequent irrigation compared to other dry and hot seasonal windows.

### 2.3 Observation schedule and data analyses

The establishment of planting was followed by observations commencing in the first month. Morphological parameters were recorded before cutting, while green foliage yield, waste generated and dry matter content were recorded after each monthly cutting. Plant height (cm), number of tillers, number of leaves, leaf length (cm), leaf width (mm), stem length (cm), stem width (mm), and crop spread (cm) were measured to determine the growth and morphology of selected *A. tuberosum* accessions. Crop spread was calculated as the average diameter of the clump spread over the ground. Five plants were randomly selected and tagged in each replication. The number of leaves per plant was calculated by counting the total number of leaves from each tagged plant. The data was analyzed using basic statistics and a two-factorial randomized block design analysis of variance. The quality of green foliage cuttings was further evaluated in terms of waste-to-yield ratio and stem length-to-plant height ratio.

### 2.4 The analysis of the nutritional and biochemical profile of *A. tuberosum*

#### 2.4.1 Estimation of proximate composition

The proximate composition of the *A. tuberosum* leaves was analyzed following the standard food analysis methods described in the Association of Official Analytical Chemists. The moisture was determined using a hot air oven as per AOAC 952.08, 2016 (Cunniff and Washington, 1997), ash content was estimated by heating plant sample in a muffle furnace for about 5-6 h at 500°C (Official Methods of Analysis of AOAC, 2016; Cunniff and Washington, 1997) whereas the acid insoluble ash content was determined by dissolving ash in dilute hydrochloric acid (10% w/w) (Official Methods of Analysis of AOAC, 2016; (Cunniff and Washington, 1997), the liquid was filtered through an ashless filter paper and thoroughly washed with hot water. The filter paper was then ignited in the original dish, cooled and weighed. The crude lipid was extracted from a moisture-free sample with petroleum ether (60-80°C) in a Soxhlet apparatus for about 6-8 h. Estimation of crude fiber content in the plant materials was carried out by treating the fat and moisture-free materials with 1.25% dilute acid and 1.25% alkali followed by washing with water and ignition of the residue. The crude protein was determined using the micro Kjeldahl method (AOAC 992.23, 2016; Sarkar et al., 2020). Total fat was determined according to the acid hydrolysis method (AOAC 948.15, 2016; Cunniff and Washington, 1997), using a Soxhlet extractor at 60°C until constant weight. Dietary fiber was determined according to the enzymatic gravimetric method (AOAC 985.29, 2016; Cunniff and Washington, 1997), digested leaf samples with heat using alpha-amylase, protease, and amyloglucosidase at 60°C, respectively, then added ethanol to samples to precipitate fiber (McCleary, 2023; Prosky et al., 1985).

The total carbohydrate content was estimated as described in the method of Hedge et al. (1962) by calculation having estimated all the other fractions by proximate analysis i.e. % Available carbohydrates = 100 - [% Moisture + % Ash + % Fat +% Protein + % Fiber]. The % organic matter was calculated as per James (2013). The energy content was determined by multiplying the values obtained for protein, fat and available carbohydrate by 4.00, 9.00 and 4.00, respectively and adding up the values. Total sugar, reducing sugar, and non-reducing sugar were calculated as per the Lane and Eynon method (James, 2013) which measures sugar as reducing sugar and total sugar as invert sugar. Non-reducing sugar is determined by subtracting the total reducing sugar from the total sugar and multiplying the remainder with a 0.95 factor.

#### 2.4.2 Estimation of minerals

The analysis of the mineral composition of *A. tuberosum* leaves was done using a spectrometer as per AOAC 990.08:2023 (Szydłowska and Szłyk, 2003; Piatkowska et al., 2015; Isaac and Johnson, 1985). Plant material was taken in a pre-cleaned and constantly weighed silica crucible and heated in a muffle furnace at 400°C till there was no evolution of smoke. The crucible was cooled at room temperature in a desiccator and carbon-free ash was moistened with concentrated sulphuric acid and heated on a heating mantle till fumes of sulphuric acid ceased to evolve. The crucible with sulphated ash was then heated in a muffle furnace at 600°C till the weight of the content was constant. One gram of sulphated ash obtained above was dissolved in 100 ml of 5% hydrochloric acid (HCl) to obtain the solution ready for determination of mineral elements through atomic absorption spectroscopy (AAS) (AA 800, Perkin-Elmer, Germany). A standard solution for each element was prepared and calibration curves were drawn for each element using AAS (Indrayan et al., 2005). Total nitrogen was estimated using the modified Kjeldahl method as per IS 7219 (Bremner and Mulvaney, 1982; Sáez-Plaza et al., 2013; Sarkar et al., 2014). The total Sulphur content was estimated by inductively coupled plasma-optical emission spectrometry (ICP-OES) (iCAP PRO-X-Duo, Thermo-Scientific, MA, USA) after sample preparation with tetramethylammonium hydroxide (TMAH) (Tavares et al., 2020; Hansen et al., 2013).

### 2.5 Estimation of phenolic acids and flavonoids

#### 2.5.1 Total phenol content

The total phenolic content of the samples was quantitatively analyzed using the Folin-Ciocalteu reagent, following the procedure (Blainski et al., 2013). Gallic acid served as the standard for this assay. The absorbance of the reaction mixture was measured at a wavelength of 750 nm using a UV-Vis spectrophotometer (Model no. UV1800, Shimadzu Corporation, Japan). The phenolic content in the samples was determined by comparing the absorbance values with a standard curve prepared using known concentrations of gallic acid (1, 2, 5, 7, 10, 20 and 50 ppm). The results were expressed as milligrams of Gallic Acid Equivalent per gram of dry weight (mg GAE/g DW).

#### 2.5.2 Total flavonoid content

The total flavonoid content was determined using a modified version of the aluminium chloride colorimetric method, with quercetin as the reference standard (Sulastri et al., 2018). In this assay, the sample extracts were reacted with aluminium chloride, and the resulting complex’s absorbance was measured at 510 nm. The flavonoid content was quantified by comparing the absorbance readings against a calibration curve generated with quercetin standards by a UV-Vis spectrophotometer (Model no. UV1800, Shimadzu Corporation, Japan). The results were articulated in terms of milligrams of Quercetin Equivalents per gram of fresh weight (mg QE/g FW).

#### 2.5.3 Antioxidant activity (DPPH assay)

The antioxidant activity of the sample extracts was evaluated using the DPPH (2,2-diphenyl-1-picrylhydrazyl) radical scavenging assay (Scherer and Godoy, 2009). The procedure involved adding 50 µL of the sample extract to 1.950 mL of a DPPH solution, which was prepared in 95% methanol to achieve an initial absorbance of 0.8 to 0.9 at 515 nm. The mixture was incubated at room temperature in the dark for 90 minutes to allow the reaction to occur. Post incubation, the decrease in absorbance was measured at 515 nm using a spectrophotometer (Model no. UV1800, Shimadzu Corporation, Japan). The antioxidant activity was quantified as the percentage of DPPH radical inhibition, calculated using the following formula:


$$\text{Percent inhibition}=\frac{\text{Absorbance of control}-\text{Absordance of sample}}{\text{Absorabnce of control }}\text{x }100$$

This percentage indicates the capacity of the sample to scavenge DPPH radicals, thereby reflecting its antioxidant activity

### 2.6 Estimation of vitamins

The estimation of vitamins was outsourced to an ISO/IEC 17025:2017 accredited laboratory (Food Hygiene & Health Laboratory (FHHL), Pune, India). The stock standard solutions of vitamins B1, B2, B3, B5 and B6, B7 & B9 were prepared by dissolving 25 mg of each standard in 1 ml 0.1M hydrochloric acid in a 25 ml standard volumetric flask. For the preparation of standard stock solutions of vitamin B9 and B2, 25 mg of each standard were dissolved in one ml 0.1 M sodium hydroxide in a 25 ml standard volumetric flask. The standard solution was stored in amber-glass bottles in the refrigerator at 4°C. The working standards were prepared by diluting with phosphate buffer (1 M, pH 5.5). For the preparation of the sample solution, plant materials were washed with distilled water. The washed plant materials were cut into very small pieces, frozen in liquid nitrogen and kept at 20°C until analysis. One gram of each of the freeze-dried samples was soaked in 10 ml water and extracted with 1 ml 0.1 M NaOH and 10 ml phosphate buffer (1 M, pH 5.5) was added to it and kept in the dark for 24 hours. The solution was first filtered through a Whatman No. 1 filter paper and the resulting filtrate was taken in a 25 ml volumetric flask and the solution was topped up to the mark with HPLC grade water. The sample solution was filtered through a 0.45 µm membrane filter before injection into the LC system. The stock solutions of the sample were kept in a refrigerator for further use. The chromatographic analysis was carried out following the method described by Seal et al. (2017) with minor modifications on an LC-MS/MS system (API 5500-MS system connected to Exion LC, Sciex, Foster City, CA, USA). The mobile phase contains acetonitrile (Solvent A) and aqueous trifluoro acetic acid (TFA, 0.01% v/v) (Solvent B), the column was thermostatically controlled at 220°C and the injection volume was kept at 20 ml. A gradient elution was performed by varying the proportion of solvent A to solvent B. The total analysis time per sample was 35 minutes. Estimation of vitamin B4 was done using LC-MS (API 5500-MS system connected to Exion LC, Sciex, Foster City, CA, USA) as per AOAC official method 2012.18 (Martin et al., 2013).

To quantify fat-soluble vitamins A, E, and K in *A. tuberosum* leaves the AOAC official method 2014.02 was followed (Giménez and Martin, 2018). Freshly harvested leaves were immediately submerged in liquid nitrogen to halt enzymatic degradation and stored at -80°C. For analysis, the frozen leaves were pulverized under liquid nitrogen and a weighed portion was extracted using a solvent system optimized for all three vitamins (methanol and buffered aqueous solution). The extract was then analyzed by UHPLC (LC 2030C-3D-Plus, Shimadzu Corporation, Japan) with a PDA detector for vitamin A and fluorescence detector for vitamins E and K. Separation was achieved using a suitable chromatographic column and optimized mobile phase conditions as outlined in the AOAC method.

For estimation of Vitamin C (Ascorbic acid), two grams (2 g) of the sample was dissolved in 50 ml of a 20% metaphosphoric acid-acetic acid mixture. After filtration, 10 mL of the filtrate was titrated with a solution of 2,6-dichlorophenolindophenol (DCPIP) at 0.5 g/L until a persistent pink coloration was observed. The final content of vitamin C was calculated using the following formula:


$$\text{Vitamin C }\left(\text{mg}/100\text{g}\right)=\frac{C_{DCPIP}\times Veq\times 5\times 100}{w}$$

Where:

Vitamin C: Vitamin C content (mg/100 g),

∁_DCPIP_​: Concentration of DCPIP solution (0.5 g/L),

V_eq_​: Volume (mL) of DCPIP used for titration,

W: Weight (g) of *A. tuberosum* sample.

### 2.7 Estimation of other biochemical & physiochemical properties and compounds

The major Sulphur active compounds viz. Allicin, AMThs (Allyl methyl thiosulphinate) and ATPThs (Allyl trans-1-propenyl thiosulphinate) were estimated by Liquid Chromatography Tandem Mass Spectrometry (LC-MS/MS) (Thangasamy et al., 2018; Singh et al., 2020). commercially available certified reference standards alliin (S-alliin-L-cysteine sulphoxide) having purity >90% and MS grade solvents were used. Alliin stock solution was prepared at a specific concentration and stored at -20°C. Allicin standard solution was generated from the alliin stock using a commercially available or purified alliinase enzyme, following a conversion process with centrifugation and storage at -80°C. Approximately 100 g of leaf sample was chopped, and crushed and a specific weight was extracted with water following a modified version of the method by Khar et al. (2011). The final extract was filtered and injected into the LC-MS/MS system (API 5500 Q-trap LC-MS/MS, AB Sciex, Canada) for analysis with preoptimized instrument parameters (Singh et al., 2020). The analytical method was validated according to a specific guideline, assessing linearity, limit of detection, limit of quantification, and precision.

The total soluble solids were measured using a refractometer as per the AOAC 932.12.2023 and the results were reported in °B. The pH was estimated using a pH meter at 10% solution as per ISO 11289:1993. Titrable acidity was measured by titration with 0.1N NaOH using phenolphthalein indicator as per Appendix A of IS 13242:2018.

### 2.8 Experimental design and statistical analysis

The study employed a factorial randomized block design to evaluate the effects of age windows (eight windows each of three months’ unit) and *A. tuberosum* accessions (three) on production performance. Descriptive statistics were used to summarize the collected data (e.g., mean, standard deviation). A two-factor analysis of variance (ANOVA) was then conducted to determine the statistical significance of the main effects (age windows and accessions) and their interaction effect on the measured parameters. This analysis helps identify if there are significant differences in the responses (e.g., plant height, yield) between the different age windows and accessions, and if these differences depend on each other. The nutritional and biochemical profile of the superior *Allium tuberosum* line, i.e. *A. tuberosum* CGN Kaz was represented with a simple mean value along with standard error.

## 3 Results

The performance of underutilized alliums was studied over two years, analyzing production data to assess the effects of age, season, and inherent characteristics on growth, yield, and quality.

### 3.1 Crop growth, productivity and produce quality

#### 3.1.1 Morphology

The morphological traits of *Allium tuberosum* accessions exhibited significant differences across various age windows, particularly in plant height, tiller number, crop spread, leaf width, and stem length (**Table 1**). The plant height ranged from 21.53 cm to 34.61 cm, depending on the accession and age window, with AT1 showing the greatest height at 270 DAT (34.61 cm). Seasonal factors had a more profound impact on plant height than the age window alone, as indicated by higher values recorded during periods of bright sunshine hours i.e. April to June (age window 90 DAT to 180 DAT and 450 DAT to 540 DAT). The tiller counts also increased significantly with age, starting from approximately 2 tillers at 90 DAT and reaching over 43 tillers per plant at 720 DAT, with AT1 consistently producing the highest number of tillers. In terms of crop spread, AT1 exhibited the widest spread of 18.52 cm at 720 DAT, followed by AT3 and AT2.

**Table 1:** Growth and morphology of *A. tuberosum* accessions at Western Ghat condition.

Age window	090DAT	180DAT	270DAT	360DAT	450DAT	540DAT	630DAT	720DAT	Mean
Plant height									
AT1	22.03 (1.04)	30.85 (1.52)	34.61 (2.10)	33.01 (0.83)	27.79 (1.45)	30.49 (1.45)	32.90 (1.11)	22.90 (1.16)	29.32a
AT2	22.24 (1.14)	29.99 (2.23)	31.95 (0.62)	31.74 (1.28)	26.83 (1.14)	29.90 (1.67)	32.01 (1.01)	22.85 (0.62)	28.44b
AT3	21.53 (0.35)	30.38 (0.45)	32.80 (2.07)	32.49 (0.85)	25.73 (2.97)	27.98 (3.68)	31.48 (2.10)	24.21 (2.21)	28.32b
Mean	21.93a	33.12a	32.41a	32.13b	30.41b	29.45c	26.783d	23.32e	
	Variety	Age window	Variety × Age window						
SEm	0.2176	0.3553	0.6154						
LSD	0.6112	0.9979	1.7285						
CV	4.7955								
No. of tillers									
AT1	2.622 (0.101)	6.17 (0.355)	11.76(0.455)	25.84(0.996)	27.762 (1.072)	32.874 (1.25)	36.712 (1.37)	43.564 (1.675)	23.414a
AT2	2.366 (0.099)	5.944 (0.245)	11.27(0.452)	25.18 (0.955)	27.16 (0.838)	32.094 (1.221)	35.392 (1.359)	42.542 (1.635)	22.891b
AT3	2.634 (0.096)	5.964 (0.213)	12.028 (0.448)	25.766 (0.978)	25.798 (0.991)	32.1 (1.232)	35.718 (1.364)	43.12 (1.643)	22.744b
mean	2.541h	6.026g	11.689f	25.596e	26.907d	32.356c	35.941b	43.075a	
	Variety	Age window	Variety x Age window						
SEm	0.0877	0.1432	0.248						
LSD	0.2463	0.4022	0.697						
CV	2.4089								
Crop spread									
AT1	3.75 (0.14)	5.52 (0.21)	8.98 (0.34)	12.79 (0.49)	15.49 (0.59)	16.7 (0.64)	17.55 (0.67)	18.52 (0.71)	12.41a
AT2	3.14 (0.12)	5.48 (0.21)	8 (0.31)	12.47 (0.48)	15.11 (0.58)	15.78 (0.6)	17.17 (0.66)	17.86 (0.69)	12.19b
AT3	3.63 (0.14)	5.92 (0.23)	8.53 (0.33)	12.55 (0.48)	15.31 (0.59)	16.35 (0.63)	17.29 (0.66)	17.97 (0.69)	11.88c
mean	3.51h	5.64g	8.5f	12.6e	15.3d	16.28c	17.34b	18.12a	
	Variety	Age window	Variety x Age window						
SEm	0.0325	0.0531	0.092						
LSD	0.0913	0.1491	0.258						
CV	1.6914								
No of leaves									
AT1	4.11 (0.16)	5.28 (0.21)	4.22 (0.16)	4.51 (0.18)	4.22 (0.17)	5.17 (0.19)	4.45 (0.18)	4.34 (0.17)	4.54a
AT2	4.25 (0.16)	5.24 (0.2)	4.29 (0.15)	3.99 (0.17)	3.96 (0.15)	5.34 (0.34)	4.12 (0.16)	3.83 (0.14)	4.38ab
AT3	4.38 (0.19)	5.36 (0.21)	4.64 (0.19)	4.43 (0.18)	3.47 (1.28)	3.47 (1.28)	4.49 (0.28)	4.26 (0.16)	4.31b
mean	4.25c	5.3a	4.38bc	4.31c	3.88d	4.66b	4.35c	4.14cd	
	Variety	Age window	Variety x Age window						
SEm	0.0684	0.1058	0.1833						
LSD	0.182	0.2972	0.5148						
CV	9.2961								
Leaf width									
AT1	7.61 (0.31)	8.37 (0.34)	6.39 (0.26)	6.64 (0.27)	8.3 (0.33)	8.49 (0.35)	6.15 (0.24)	8.28 (0.31)	7.53a
AT2	7.13 (0.29)	7.74 (0.31)	5.73 (0.25)	6.21 (0.24)	5.93 (0.23)	8.2 (0.33)	5.78 (0.22)	8.09 (0.32)	7.19b
AT3	7.6 (0.3)	8.13 (0.32)	6.15 (0.24)	6.37 (0.23)	6.49 (0.25)	8.3 (0.32)	6.34 (0.32)	8.18 (0.31)	6.85c
mean	7.447d	8.08c	6.091g	6.408f	6.905e	8.329a	6.091g	8.183b	
	Variety	Age window	Variety x Age window						
SEm	0.0157	0.0257	0.0445						
LSD	0.0441	0.0722	0.125						
CV	1.3851								
Stem length									
AT1	2.3 (0.1)	4.39 (0.19)	7.33 (0.31)	5.58 (0.23)	4.67 (0.2)	5.31 (0.22)	7.81 (0.3)	4.74 (0.2)	5.27a
AT2	3.37 (0.35)	4.18 (0.15)	7.18 (0.29)	4.74 (0.2)	4.22 (0.18)	4.52 (0.17)	7.78 (0.35)	4.51 (0.26)	5.1b
AT3	2.9 (0.13)	4.14 (0.17)	6.43 (0.25)	5.4 (0.23)	4.34 (0.18)	5.13 (0.2)	7.9 (0.73)	4.56 (0.2)	5.06b
mean	2.857h	4.237g	6.983b	5.241c	4.411f	4.988d	7.831a	4.601e	
	Variety	Age window	Variety x Age window						
SEm	0.0271	0.0443	0.0767						
LSD	0.0761	0.1244	0.2154						
CV	3.3333								
Stem girth									
AT1	5.38 (0.19)	6.03 (0.32)	4.51 (0.1)	5.64 (0.15)	4.28 (0.25)	4.68 (0.15)	4.35 (0.19)	4.56 (0.17)	4.93a
AT2	5.13 (0.2)	6.11 (0.23)	4.1 (0.17)	5.59 (0.21)	4.09 (0.17)	4.75 (0.25)	4.25 (0.17)	4.46 (0.17)	4.81b
AT3	5.15 (0.22)	5.72 (0.28)	4.24 (0.17)	5.25 (0.21)	4.13 (0.17)	4.63 (0.17)	4.2 (0.17)	4.18 (0.19)	4.69c
mean	5.22c	5.95a	4.28f	5.49b	4.17g	4.68d	4.27f	4.4e	
	Variety	Age window	Variety x Age window						
SEm	0.0117	0.0191	0.033						
LSD	0.0329	0.0536	0.0927						
CV	1.5364								

Figures in the parenthesis show the standard deviation. The LSD represents least square values at 5%. The mean values indicated with different letters show significant difference. AT1: *A.tuberosum* All-1587, AT2: *A. tuberosum* CGN-116418 and AT3: *A. tuberosum* CGN-16373.

The variations in leaf width and stem length were similarly pronounced across different age windows. AT1 had the widest leaves at 720 DAT (8.28 cm), while the longest stem length was also observed at 720 DAT, with AT1 and AT3 recording lengths of 7.81 cm and 7.9 cm, respectively. The leaf count showed moderate differences across accessions, with AT1 consistently producing a higher number of leaves, peaking at 5.17 leaves per plant at 540 DAT. These results suggest that both genetic makeup and the age window strongly influenced the morphology of the accessions, with seasonal effects playing an equally important role in shaping plant structure, especially during windows of bright sunshine.

**Tables 2**, **3** show that statistical significance was evident for most morphological traits. The least-square difference (LSD) values from the tables confirmed that the differences in plant height, tiller count, crop spread, and other morphological parameters were indeed significant across accessions and age windows, further reinforcing the impact of age and genetic variation on plant structure. This significance emphasizes the necessity of considering both genetic factors and environmental conditions when evaluating the morphology of *A. tuberosum*.

**Table 2:** Green foliage yield and related parameters of *A. tuberosum* accessions at Western Ghat condition.

Age window	090DAT	180DAT	270DAT	360DAT	450DAT	540DAT	630DAT	720DAT	mean
Green foliage yield									
AT1	4.56 (0.18)	8.98 (0.34)	14.23 (0.55)	18.95 (0.73)	24.17 (0.93)	26.69 (1.02)	28.68 (1.1)	30.75 (1.18)	19.63a
AT2	3.79 (0.14)	8.53 (0.33)	13.44 (0.52)	18.31 (0.7)	21.89 (0.84)	26.07 (1)	28.13 (1.08)	29.67 (1.14)	18.73b
AT3	4.13 (0.16)	8.32 (0.32)	14.05 (0.54)	18.17 (0.7)	22.8 (0.88)	25.08 (0.96)	28.02 (1.08)	29.21 (1.12)	18.72b
Mean	4.16h	8.61g	13.9f	18.48e	22.95d	25.95c	28.28b	29.88a	
	Variety	Age window	Variety ×Age window						
SEm	0.0547	0.0893	0.1546						
LSD	0.1536	0.2508	0.4342						
CV	1.8169								
Waste									
AT1	0.5 (0.02)	1.08 (0.04)	2.71 (0.11)	2.65 (0.1)	2.9 (0.11)	2.67 (0.1)	6.38 (0.25)	5.23 (0.2)	3.01a
AT2	0.3 (0.01)	1.02 (0.04)	2.55 (0.1)	2.56 (0.1)	2.63 (0.1)	2.61 (0.1)	6.19 (0.23)	5.04 (0.2)	2.88b
AT3	0.43 (0.02)	1(0.04)	2.67 (0.1)	2.54 (0.1)	2.74 (0.11)	2.51 (0.1)	6.16 (0.24)	4.96 (0.19)	2.86b
mean	0.41g	1.03f	2.64d	2.59e	2.75c	2.6de	6.25a	5.08b	
	Variety	Age window	Variety × Age window						
SEm	0.0112	0.0182	0.0316						
LSD	0.0315	0.0511	0.0888						
CV (%)	2.42								
Dry matter									
AT1	11.42 (0.51)	13.01 (0.38)	9.51 (0.28)	10.61 (0.3)	10.76 (0.65)	13.3 (0.85)	10.36 (1.03)	10.44 (0.27)	11.18a
AT2	10.59 (0.47)	12.06 (0.35)	8.82 (0.25)	9.83 (0.28)	9.98 (0.6)	12.33 (0.79)	9.61 (0.96)	9.68 (0.25)	10.47b
AT3	10.69 (0.48)	12.17 (0.35)	8.9 (0.26)	9.93 (0.28)	10.07 (0.61)	12.45 (0.79)	9.7 (0.96)	9.88 (0.26)	10.36b
mean	10.9b	12.41a	9.08d	10.12c	10.27c	12.7a	9.89c	10c	
	Variety	Age window	Variety x Age window						
SEm	0.0833	0.136	0.2356						
LSD	0.234	0.382	0.6617						
CV (%)	4.93								

Figures in the parenthesis show the standard deviation. The LSD represents least square values at 5%. The mean values indicated with different letters show significant difference. AT1: *A.tuberosum* All-1587, AT2: *A. tuberosum* CGN-116418 and AT3: *A. tuberosum* CGN-16373.

**Table 3:** Eigenvectors.

Variables	PC1	PC2	PC3
Plant height	0.122	0.345	0.503
No of tillers	0.319	-0.371	0.062
No of leaves	-0.127	0.055	0.599
Leaf width	-0.167	-0.480	0.212
Stem length	0.322	0.286	0.260
Stem girth	-0.292	0.051	0.302
Crop spread	0.328	-0.347	0.078
Green foliage	0.336	-0.338	0.104
Waste	0.382	-0.108	0.074
Stem to leaf ratio	0.360	0.110	0.038
Waste to yield ratio	0.327	0.272	0.054
Dry matter %	-0.218	-0.305	0.392
Statistics			
Standard deviation	2.471	1.540	1.301
Proportion of Variance	0.509	0.198	0.141
Cumulative Proportion	0.509	0.707	0.848
Eigen Values	6.108	2.371	1.694

#### 3.1.2 Crop growth

The crop growth patterns over the study period, specifically regarding tillering, crop spread, and dry matter content, showed steady increases with age across all accessions. Tillering, which began with an average of around 2.5 tillers at 90 DAT, grew progressively to over 43 tillers by 720 DAT. AT1 consistently led in tiller production across all age windows, although the other accessions (AT2 and AT3) followed closely behind. Similarly, crop spread increased with age, starting at 3.75 cm at 90 DAT and reaching a maximum of 18.52 cm at 720 DAT for AT1, while AT3 and AT2 showed slightly smaller, yet significant, values. Dry matter content was influenced by both crop age and seasonal variations. The highest dry matter yields were recorded during the 90–180 DAT and 450–540 DAT windows, coinciding with periods of bright sunshine and elevated temperatures. AT1 maintained the highest average dry matter content (11.18%), while AT2 and AT3 trailed behind slightly. This seasonal trend demonstrates the strong interplay between environmental conditions and crop physiology, with the brightest sunshine hours contributing to higher dry matter accumulation.

The significant differences between accessions, as shown by the LSD values, underscore the importance of considering both genetic traits and environmental conditions when evaluating the crop growth of *A. tuberosum*. The growth patterns observed suggest that tillering and dry matter content are not only dependent on age but also heavily influenced by external factors such as sunshine hours and temperature, further validating the role of environmental variables in crop development.

#### 3.1.3 Yield and quality of harvest

The quality of the harvest was assessed through a range of parameters, including green foliage yield, waste generated, the waste-to-yield ratio, and the stem-to-leaf ratio. Fresh foliage yield increased significantly with crop age across all accessions, with AT1 producing the highest overall yield. In the 630-720 DAT window, AT1 yielded 30.75 tons/ha, whereas AT2 and AT3 produced slightly lower yields of 29.67 tons/ha and 29.21 tons/ha, respectively. Over the two years, AT1 accumulated a total yield of 157.01 tons/ha, significantly higher than the other accessions, illustrating its superior productivity. However, the higher productivity also led to a proportional increase in waste generation, with AT1 producing 6.38 tons/ha of waste in the 540-630 DAT window, while AT2 and AT3 followed closely behind. The waste-to-yield ratio was highest during the 180-270 and 540-630 DAT windows, coinciding with the monsoon season, which negatively impacted foliage quality.

**Figure 2:**
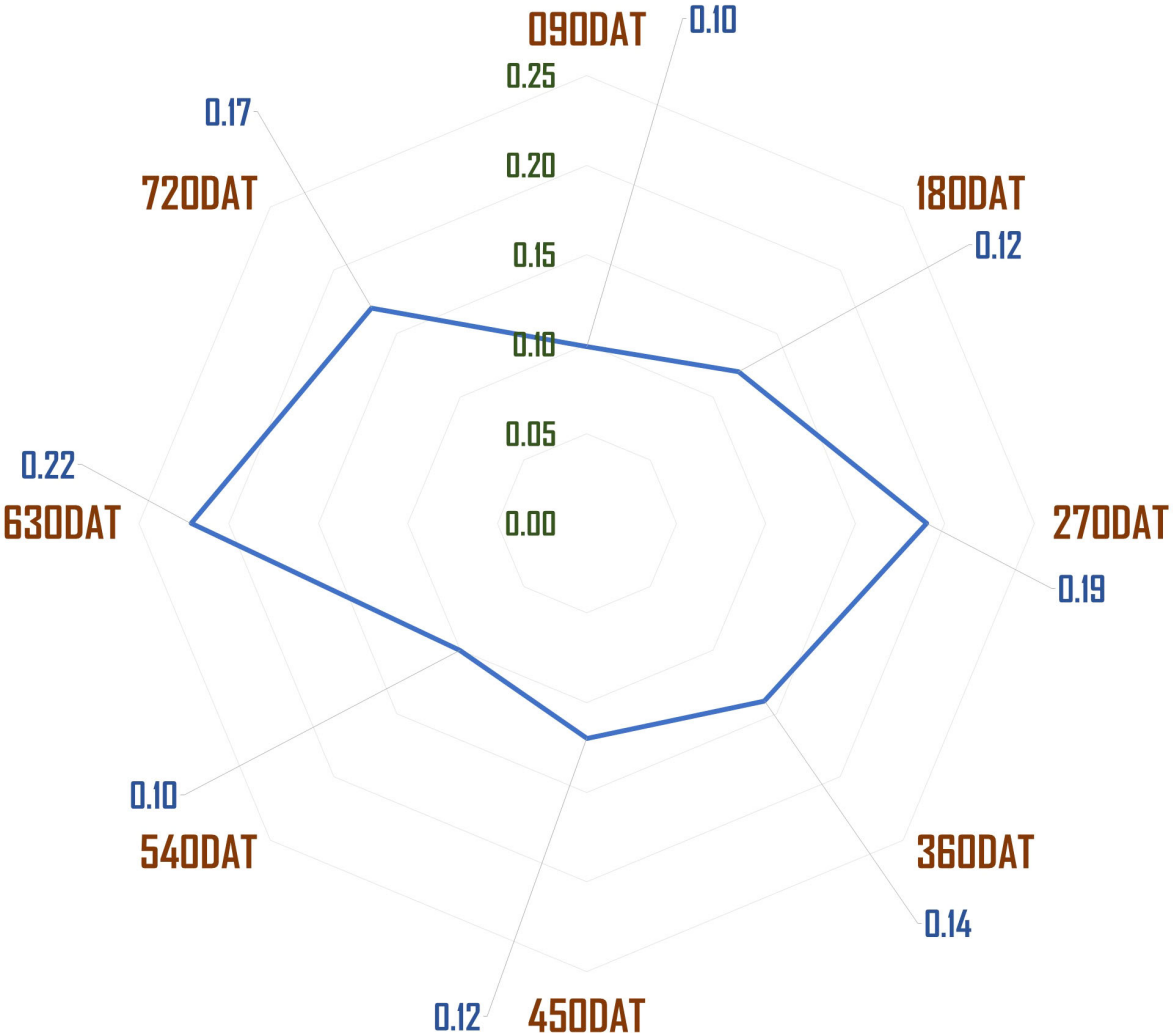
Stem length to plant height ratio across different age windows.

**Figure 3:**
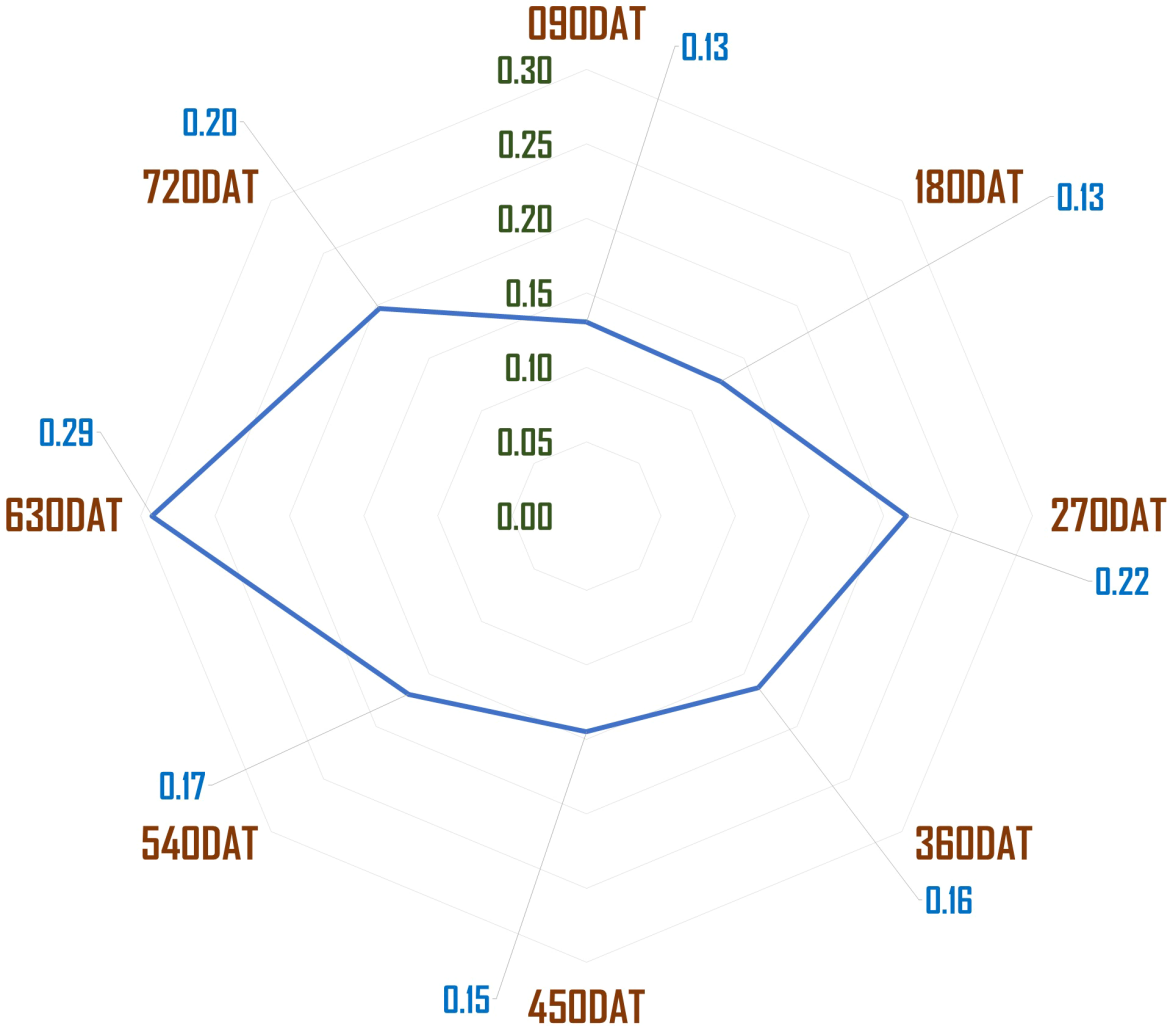
Waste generated to green foliage yield ratio across different age windows.

The stem-to-leaf ratio, which served as a critical measure of harvest quality, indicated that a higher ratio corresponded to poorer quality, as the crop is primarily cultivated for its leaves. Moreover, the results suggest that seasonal variations, particularly during monsoon windows, had a profound effect on yield quality. The stem-to-leaf and waste-to-yield ratios were notably higher during these periods, confirming that environmental conditions played a critical role in determining harvest quality. These findings, combined with the significant LSD values, further validate the hypothesis that age, genetics, and seasonality collectively influence both yield and its quality (**Figures 2**, **3**).

### 3.2 The association of different growth and yield parameters

**Figure 4:**
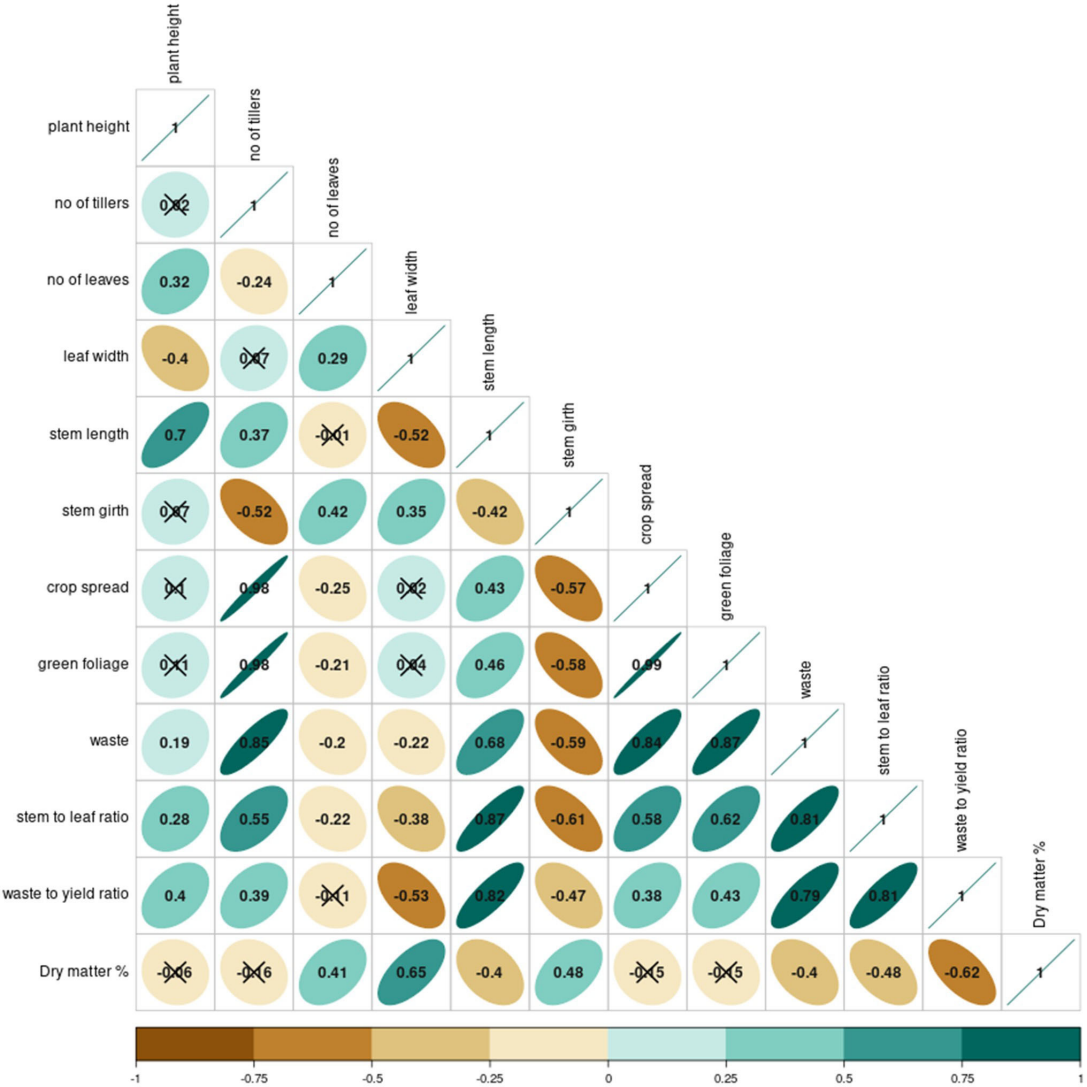
Correlations between different yield and morphological parameters of *A. tuberosum*.

The correlations between different morphological and yield parameters of *A. tuberosum* as given in **Figure 4** reveal intriguing results. The green foliage yield was found positively correlated with the tillering. As these tillers are added with the age of the plant, it widens the crop spread which is supported by a highly positive correlation between crop spread with yield (r = 0.89) and the tillers (r = 0.98). However, this crop spread increment was also associated with inferior quality traits i.e. the waste (r = 0.89) and stem-to-leaf ratio (r = 0.58). This hints at a possible trade-off i.e. increased vegetative growth might come at the cost of generating more stem and inferior quality harvest. This connection is further strengthened by the strong positive correlation between waste and waste-to-yield ratio (waste-waste to yield ratio: r = 0.813, green foliage yield-waste to yield ratio: r = 0.836). Interestingly, the table reveals some counterpoints. A negative correlation of both leaf width and stem girth with the number of tillers (leaf width-tillers: -0.237, stem girth-tillers: r = -0.523) was observed.

In addition to the aforementioned correlations, other interesting patterns emerged from the correlogram. The leaf length showed a positive yet moderate correlation with green foliage yield (r = 0.55), implying that longer leaves contribute somewhat to the overall yield, though not as significantly as tillering. On the contrary, the stem-to-leaf ratio was negatively correlated with yield quality parameters like leaf width (r = -0.42), indicating that a higher stem-to-leaf ratio might reduce leaf quality, potentially due to a thicker, less desirable stem structure. Moreover, the waste-to-yield ratio was inversely related to both leaf length (r = -0.31) and leaf width (r = -0.36), reinforcing that plants with thinner or shorter leaves might generate more waste relative to the total yield, pointing to an indirect relationship between morphological attributes and overall yield efficiency. These additional correlations highlight the complexity of the trade-offs between vegetative growth and yield quality in *Allium tuberosum*.

### 3.3 The principal component analysis

**Figure 5:**
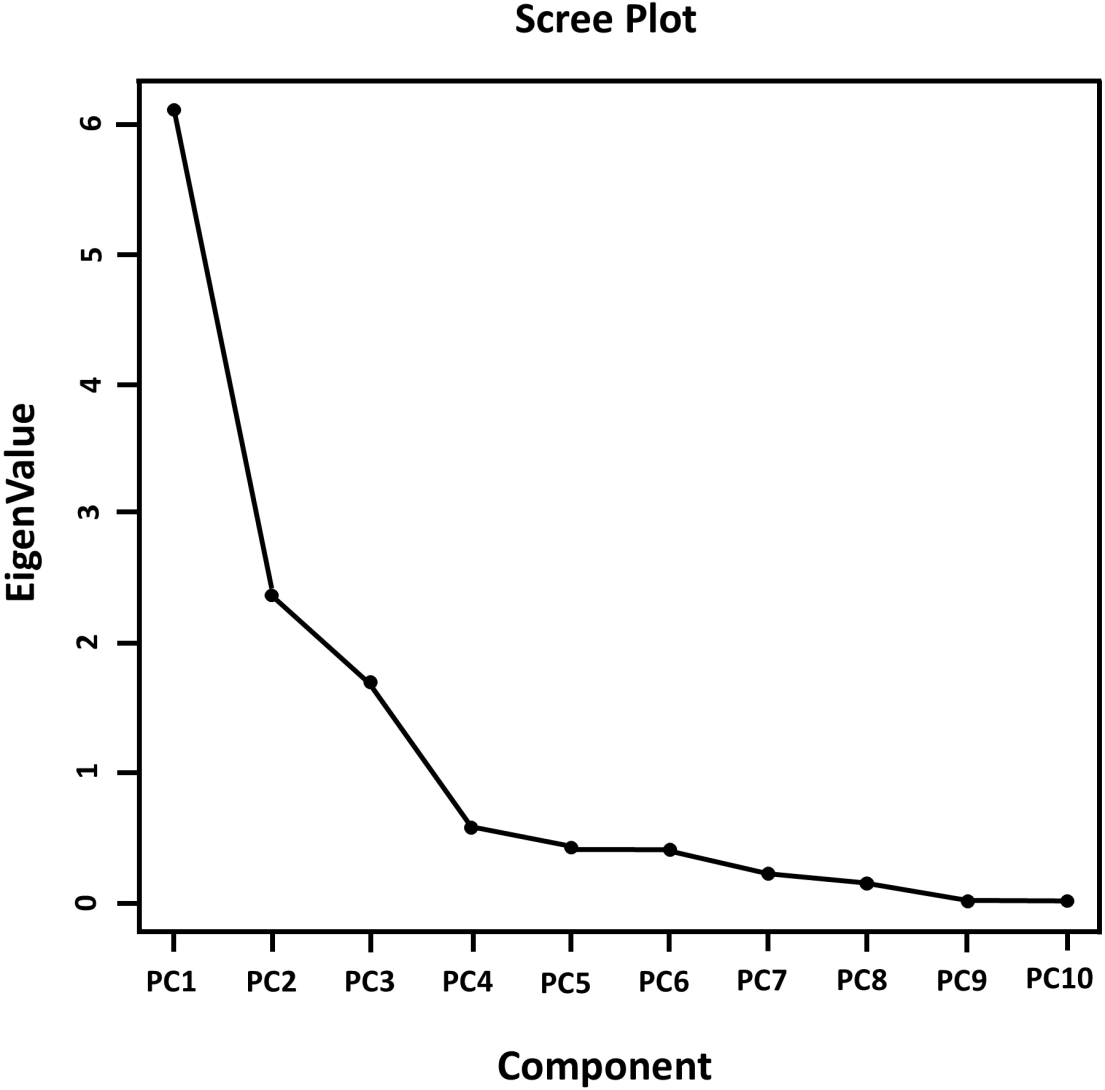
Scree plot component vs Eigenvalues.

**Figure 5** shows that the flat portion of the curve begins at component 4, indicating we should use the first three principal components. The first three principal components have eigenvalues greater than 1 (**Table 3**). The eigen analysis also indicated that PC1, PC2 and PC3 accounted for 50.9%, 19.8% and 14.1% of the variance, respectively. These principal components accounted for 84.8% of the total variability.

**Figure 6:**
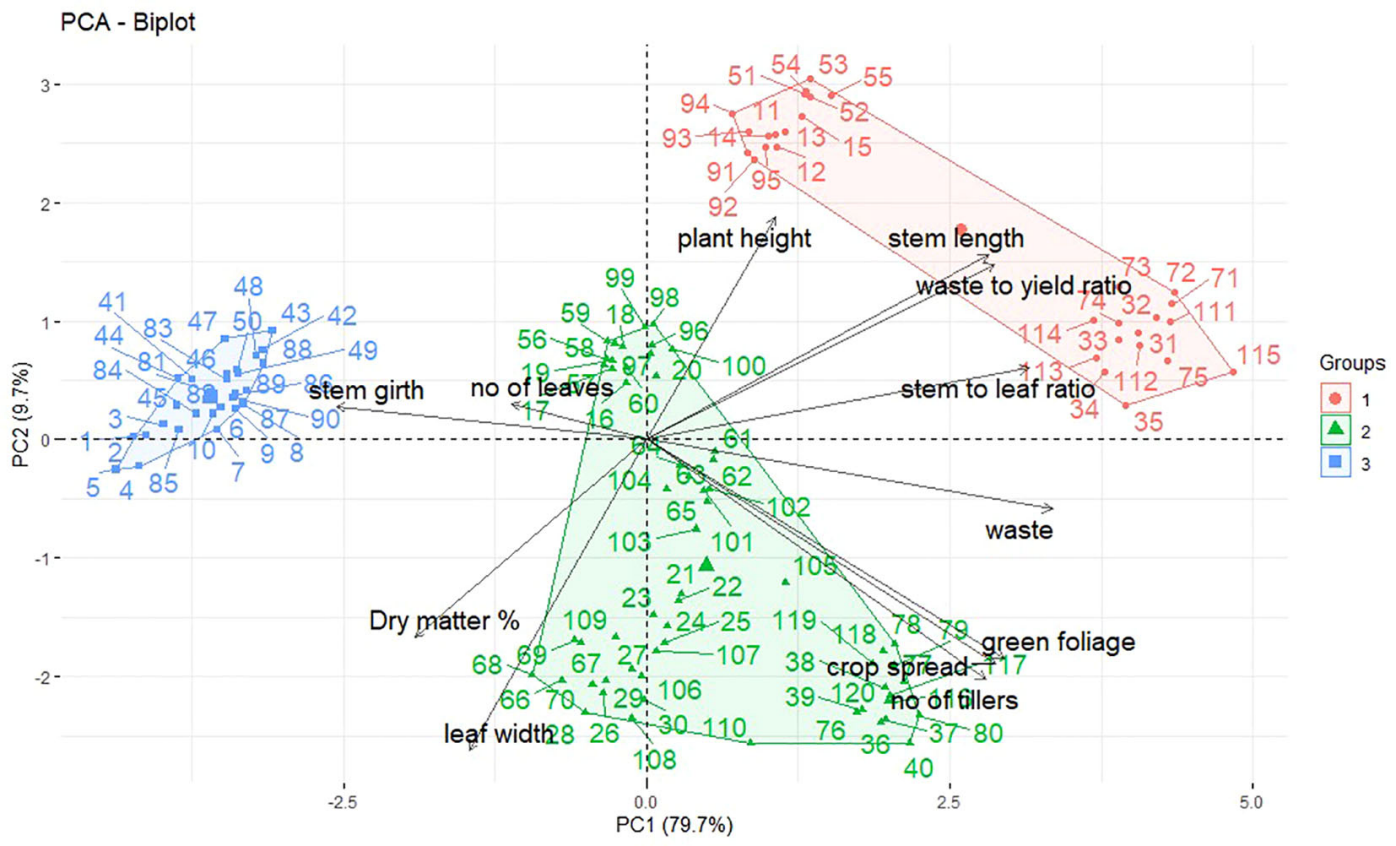
Principal component analysis- Biplot PC1 vs PC2 based on the 12 characters studied.

The coefficient of the principal component (Eigenvectors) (**Table 2**) revealed that the first principal component had large positive associations with waste, stem-to-leaf ratio, green foliage yield, crop spread, waste-to-yield ratio, stem length and the number of tillers, so this component primarily measures continuing stability at 630DAT. The second component had large negative associations with dry matter %, green foliage, crop spread, number of tillers and leaf width, so this component primarily measures yield at 720DAT. The third component had positive associations with the number of leaves, stem girth, plant height, and dry matter %, so this component mostly measures steady variability at 90DAT and 180DAT. The principal component analysis - biplot (PC1 vs PC2) visually shown in **Figure 6**.

### 3.4 The Nutritional and biochemical profile of *A.tuberosum* All-1587

#### 3.4.1 The proximate analysis & mineral composition

The proximate analysis of *A.tuberosum* All-1587 (AT1) revealed a high moisture content of 88.94% in the fresh leaf cuttings (**Table 4**). This translates to a low caloric density of 36.13 Kcal per 100g. Interestingly, this moisture content is slightly higher than reported by Pooja (2021), suggesting latent variations based on cultivation practices. Rest, the ash content of 1.47 g per 100g, with a minimal acid-insoluble fraction (0.04 g), indicates the presence of dietary minerals. The low-fat content (0.85 g per 100g) and the lower cholesterol (<0.50 g per 100g) of AT1 are promising, especially considering the commendable ratio of unsaturated to saturated fats. The presence of a notable amount of dietary fiber (2.56 g per 100g) and protein (3.10 g per 100g) in AT1 is important. Both, the protein and dietary fiber content are quite higher than the A. cepa bulbs (1.1 g and 1.7g per 100g respectively) (Bhattacharjee et al., 2013; Mahmood et al., 2021).

**Table 4:** Nutritional and biochemical profile of *A. tuberosum* Kazakhsthan CGN-1587.

Serial No.	Particular	Estimation
Proximate analysis		
1	Moisture	88.94 ± 7.55 Kcal/100g
2	Ash Content	1.47 ± 0.12 g/100g
3	Acid insoluble ash	0.04 ± 0.0028 g/100g
4	% Organic Matter	9.59 ± 0.81 g/100g
5	Total Fat	0.85 ± 0.07 g/100g
6	Saturated Fat	0.14 ± 0.01 g/100g
7	Unsaturated Fat	0.71 ± 0.06 g/100g
8	Cholesterol	<0.5 ± g/100g
9	Protein	3.1 ± 0.26 g/100g
10	Total Carbohydrates	5.3 ± 0.45 g/100g
11	Dietary Fiber	2.56 ± 0.22 g/100g
12	Total sugar	2.15 ± 0.18 g/100g
13	Energy	36.13 ± 3.07 Kcal/100g
Mineral composition		
14	N	5608.32 ± 475.88 mg/kg
15	P	691.68 ± 58.69 mg/kg
16	K	5355.3 ± 454.41 mg/kg
17	S	2484.97 ± mg/kg
18	Mg	569.39 ± 48.31 mg/kg
19	Mn	5.62 ± 0.48 mg/kg
20	Si	19.98 ± 1.7 mg/kg
21	Se	42.17 ± 5.18 mg/kg
22	Zn	53.42 ± 4.53 mg/kg
23	Cu	16.2 ± 1.37 mg/kg
24	Fe	397.46 ± 33.73 mg/kg
25	Ca	1126.99 ± 95.63 mg/kg
26	Na	14.72 ± 1.25 mg/kg
27	B	1.93 ± 0.16 mg/kg
Vitamin profile		
28	A (Retinol)	3.15 ± 0.27 mg/kg
29	B1 (Thiamine)	0.309 ± 0.03 mg/kg
30	B2 (Riboflavin)	5.27 ± 0.45 mg/kg
31	B3 (Niacin)	4.87 ± 0.41 mg/kg
32	B4 (Adenine)	264.45 ± 22.44 mg/kg
33	B5 (Pantothenic Acid)	0.91 ± 0.08 mg/kg
34	B6 (Pyridoxine)	0.053 ± 0.0045 mg/kg
35	B7 (Biotin)	<0.2 mg/kg
36	B9 (Folates)	<0.2 mg/kg
37	C (Ascorbic acid)	18.12 ± 3.35 mg/100g
38	E (Tocopherol)	5.38 ± 0.46 mg/kg
39	K (Phytonadione)	85 ± 7.21 mcg/kg
Biochemical properties		
40	pH (10% solution)	6.7 ± 0.57
41	Titrable acidity	0.36 ± 0.03 g/100g
42	TSS	7.93 ± 0.67°Brix
43	Total phenolic compounds	1.187 ± 0.1 mg GAE/g
44	Reducing Sugar	1.63 ± 0.14 mg/100g
45	Non-reducing Sugar	0.49 ± 0.04 mg/100g
46	Antioxidant Activity	64.45 ± 5.47% RSA DPPH
47	Allicin	18.16 ± 1.58 mg/kg
48	AMThs (Allyl methyl thiosulphinate)	301.55 ± 25.59 mg/kg
49	ATPThs (Allyl trans-1-propenyl thiosulphinate)	4.41 ± 0.37 mg/kg
50	Flavonoid (mg/g)	3.19 ± 0.27 mg/g

*Values are given as ± standard error from the mean.

The analysis of AT1 reveals a rich mineral profile dominated by essential macro-elements. Potassium (5355.30 mg/kg), followed closely by phosphorous (691.68 mg/kg), calcium (1126.99 mg/kg) and magnesium (569.39 mg/kg). In the macro-elements, a noteworthy aspect is the substantial amount of Sulphur (2484.97 ± 104 mg/kg) present in AT1. This aligns with the characteristic pungent aroma of Alliums, attributed to various volatile Sulphur compounds (Abe et al., 2020). Similarly, as the assimilation of selenium is carried out through the metabolic absorption route of sulfur (Barak and Goldman, 1997) the concentration of Se was found 42.17 mg/kg which is in line with the previous findings (Pilon-Smits et al., 2009). The presence of essential micronutrients like manganese (5.62 mg/kg), zinc (5.34 mg/kg), copper (1.62 mg/kg), and iron (39.74 mg/kg) further strengthen the nutritional value of AT. Additionally, the low sodium content (14.72 mg/kg) was also reported. The B, Cu and Zn content was comparatively lower in concentration than the *A. cepa* leaves as reported by Ferreira et al. (2022).

#### 3.4.2 The vitamin profile

The vitamin profile of *A tuberosum* (AT1) reveals a captivating interplay of water-soluble and fat-soluble vitamins, impacting human health (**Table 4**). AT stands out for its particularly high content of vitamin B4, also known as choline (264.45 mg/kg) which is approximately four times more than in *A. cepa* bulb as reported by Pareek et al. (2017). While the content of these B vitamins is not exceptionally high, their combined presence contributes to the overall nutritional value of AT1. Interestingly, pyridoxine (vitamin B6) is present in very low quantities (0.053 ± 0.0045 mg/kg), suggesting that AT may not be a significant dietary source for this particular vitamin. The presence of vitamin C (18.12 mg/100g) highlights AT1’s contribution to immune function and antioxidant activity (Carr and Maggini, 2017). This vitamin C quantity is quite less than previous reports where Żurawik and Jadczak (2008) reported 43.0 - 56.1 mg/100g of L-ascorbic acid in the leaves *A. tuberosum*. The presence of vitamin E (5.38 mg/100g) further strengthens the antioxidant properties of AT1 (Traber and Atkinson, 2007). Vitamin E helps to protect cells from free radical damage, which has been linked to various chronic diseases (Traber and Atkinson, 2007). While vitamin K content is relatively low (85.00 mcg/100g), it can contribute modestly to blood clotting and bone health (Shearer and Newman, 2008). The limited detection of folates (vitamin B9) and biotin (vitamin B7) suggests that AT1 may not be a primary source of these essential nutrients.

#### 3.4.3 The biochemical properties

The reported biochemical properties of AT1 shed light on some interesting aspects that can influence its sensory characteristics, preservation, and culinary applications (**Table 4**). A slightly acidic pH of 6.7 in a 10% AT solution indicated its weakly acidic nature (Sánchez-Rodríguez et al., 2019). Further investigation revealed the presence of organic acids (0.36 g/100g) within AT1, evident from the titrable acidity value. The total soluble solids (TSS) measured at 7.93°Brix signifies the concentration of soluble solids present in AT1 which is lower than some previous reports i.e. 8.6°Brix as reported by Pooja (2021). This TSS content is at par with that of Welsh onion leaves (7.51 ± 0.31°Brix) and lower than A. cepa leaves (5.87 ± 0.18) as reported by Masahiro Yuasa et al. (2022).

The biochemical profile of *A. tuberosum*, revealed a promising composition with various health-promoting compounds (**Table 4**). The presence of a significant amount of total phenolics (1.187 mg GAE/g fresh weight basis) suggests strong antioxidant property (Bindon et al., 2010). This is further supported by the high free radical scavenging activity (64.45% RSA DPPH). These findings align with observations in other *Allium* species for their antioxidant properties (Rahman, 2007). Interestingly, *A. tuberosum* displayed a unique profile of organosulfur compounds. While allicin, the characteristic pungent compound of garlic (*A. sativum*), was present at relatively low levels (16.60 mg kg^-1^), the dominant organosulfur compound was allyl methyl thiosulfinate (AMThs) at a considerably higher concentration (269.00 mg kg^-1^). This suggests *A. tuberosum* might possess a distinct flavor and potentially different biological activities compared to garlic (Lanzotti, 2006). The presence of flavonoids (3.19 mg/g) and ascorbic acid (18.12 mg/g) adds further value to the nutritional profile of AT1.

## 4 Discussion

The two-year production study highlight the suitability of *A. tuberosum* cultivation at Western Ghats as the yield scenarios of the green leaf cuttings were comparable with the traditional growing belt. The *A. tuberosum* yielded 157.01 tons/ha in two years by monthly cuttings, which was three times higher than the reported by Wang et al. (2020). Where the *A. tuberosum* chives production in main growing regions (i.e China, Uzbekisthan, Kazakisthan, Eastern Europe and Korea) is limited by the winter low temperatures, the Western ghat conditions provide a year-round growth season with monthly cutting intervals. More importantly, the leaf purpose market of *Allium* species is majorly dominated by *A. cepa* cultivars. It faces a seasonal slag due to monsoon and high summer heat months. Comparably the yield variations in case of *A. tuberosum* are less and can be projected as alternative to later as also supported by consumer studies by Kale et al. (2024b). The yield related studies carried out for *A. tuberosum* (Rathore et al., 2020; Pooja 2021) were mostly short durational, thus this study is the first insight at Indian planes for the comparable multi-year production.

The temporal and varietal variations in *A. tuberosum* growth dynamics present compelling insights into optimizing cultivation strategies, especially when considering the intricate interaction between environmental factors and plant morphology. Seasonal fluctuations, particularly the heightened sensitivity of AT1 to photoperiod and temperature, reaffirm the species’ dependence on these external variables for growth regulation (Rathore et al., 2020). Notably, the monsoon season’s influence, leading to an exaggerated stem elongation, supports the hypothesis of light-seeking behavior in low-sunlight conditions (Golan et al., 2024). However, this adaptive elongation, while advantageous for light capture, results in a proportional reduction in stem girth and leaf width. These findings invite deeper exploration into the physiological trade-offs plants make to cope with fluctuating environmental constraints. The distinctive patterns observed in foliage yield, especially the progressive increase in tillering, reveal a double-edged sword. Although an increase in tillers is correlated with higher yields, it simultaneously escalates waste production, a phenomenon corroborated by Żurawik and Jadczak (2008) and Uddin et al. (2019). This apparent yield-waste paradox challenges conventional notions of productivity, suggesting that beyond a certain threshold, the drive for higher foliage production may compromise overall harvest quality. Producers must thus navigate this trade-off, optimizing tillering while controlling waste accumulation to balance economic returns with quality.

A critical reflection on the crop’s morphological adjustments under different seasonal windows reveals fascinating behavioral responses. For instance, the reduction in leaf width during the monsoon, despite lower light availability, contradicts the general expectation of increased leaf area in low-light environments. This anomaly suggests a possible compensatory mechanism where the plant may prioritize structural integrity over expansive leaf growth to maintain balance, a notion in line with Brand (1997) and Sladek et al. (2009) but contrast with the findings of Marenco and Reis (1998) and Sladek et al. (2009). This observation invites further investigation into the physiological underpinnings of such adaptive strategies, particularly in light-limited environments.

The results of this study establish A. tuberosum (AT1) as a nutritionally rich crop, exhibiting significant concentrations of potassium, phosphorus, and sulphur, which are vital for cardiovascular health and metabolic regulation (Schwalfenberg and Genuis, 2017; Humphries and Dart, 2015). The high sulfur content enhances its culinary appeal and suggests therapeutic applications (Tang et al., 2017). Furthermore, the presence of organosulfur compounds, particularly allyl propyl disulfide, comparable with Singh et al. (2020) other *Allium* species as reported by Rathore et al. (2020). positions AT1 as a possible mitigator of oxidative stress associated with chronic diseases (Rahman, 2007). The favorable fat profile and low caloric content of AT1 further support its role in dietary management for individuals with metabolic disorders and low calory diets (Campos, 2018; Thompkinsons et al., 2014).

Notably, the plant’s micronutrient profile includes selenium, zinc, copper, and iron, which are essential for metabolic and immune functions (Elmadfa and Meyer, 2019). The Selenium’s countable presence justifies their common assimilation through the metabolic absorption route of sulphur as reported by González-Morales et al. (2017) and Jiang et al. (2021). The vitamins present, particularly B vitamins, vitamin C, and vitamin E bolster AT1’s antioxidant and immune-supportive properties (Mehedint and Zeisel, 2013; Doseděl et al., 2021). Overall, AT1 emerges as a significant player in dietary strategies aimed at combatting chronic diseases. To optimize the benefits of AT1, further research is necessary to validate them which is crucial for translating its nutritional advantages into effective dietary interventions.

## 5 Conclusion and implication

This study offers a comprehensive analysis of the agronomic performance, yield potential, quality traits, and nutritional profile of *A. tuberosum* under the specific environmental conditions of the Western Ghats. The *A.tuberosum* All-1587 line demonstrated the highest foliage yield, making it ideal for large-scale cultivation. The outcomes of the study give a deeper understanding of the complex interactions between agronomic traits and overall crop performance. The key implications of the study are as follows:

- The study guides farmers in tailoring harvesting schedules and spatial arrangements to specific seasonal conditions for a stable and reliable yields.
- The *A.tuberosum* All-1587 line’s superior yield and quality traits suggest significant potential for vegetable supply chains and the production of value-added products.
- High bioactive compound content stages *A.tuberosum* All-1587 as an attractive ingredient for health and wellness products, contributing to nutritional security in regions with prevalent deficiencies.
- Resilience to seasonal variability, with minimal yield fluctuations, highlight *A. tuberosum*’s high adaptability to diverse environmental conditions in the region.

Developing guidelines and best practices based on these insights will help farmers adopt optimal cultivation techniques. These findings promotes sustainable and economically viable farming practices, by expanding the cultivation in unconventional zones. The findings provide valuable insights for optimizing and commercializing *A. tuberosum*, benefiting farmers, entrepreneurs, processing industries, and supply chain developers.

## Data Availability

The original contributions presented in the study are included in the article/supplementary material. Further inquiries can be directed to the corresponding authors.
