# Correction: Cultivation viability of *Allium tuberosum* L. in the Western Ghats: insights into crop dynamics, yield and quality

**DOI:** 10.3389/fpls.2026.1881329

**Published:** 2026-05-26

**Authors:** Abhishek D. Gavhane, Rajiv B. Kale, Yogesh Khade, Hem Raj Bhandari, Shivam Y. Gaikwad, Sharadveer Singh, Ahammed Shabeer T. P., Yogesh A. Garde, Kiran Khandagale, Vijay Mahajan

**Affiliations:** 1 ICAR-Directorate of Onion and Garlic Research, Pune, India; 2 ICAR-National Research Centre for Grapes, Pune, India; 3 N.M. College of Agriculture, Navsari Agricultural University, Navsari, India

**Keywords:** underutilized *Allium*, non-traditional cultivation, yield optimization, seasonal effects, Western Ghats agro-climatic region

The use of “*A. tuberosum* Kazakhstan CGN-1587” throughout the article was incorrect. The 9 instances of “*A. tuberosum* Kazakhstan CGN-1587” have been replaced with “*A. tuberosum* All-1587”.

The original version of this article has been updated.

